# Early-Onset Dropped Head Syndrome and Person-in-the-Barrel Syndrome After Radiation Therapy: Clinical, Electromyographic, and MRI Findings

**DOI:** 10.7759/cureus.28279

**Published:** 2022-08-22

**Authors:** Lisa B Shields, Vasudeva G Iyer, Jiancong Liang, Yi Ping Zhang, Christopher B Shields

**Affiliations:** 1 Norton Neuroscience Institute, Norton Healthcare, Louisville, USA; 2 Neurology, Neurodiagnostic Center of Louisville, Louisville, USA; 3 Department of Pathology, Microbiology, and Immunology, Vanderbilt University Medical Center, Nashville, USA

**Keywords:** radiation, nerve conduction study, electromyography, person-in-the-barrel, early-onset, dropped head syndrome, neurology

## Abstract

Dropped head syndrome (DHS) involves severe weakness of the neck extensor muscles causing the mandible to drop to the chest wall. Isolated neck extensor weakness is a rare complication of radiotherapy. This condition may result within a few weeks or months following radiotherapy (early-onset) or several years after radiotherapy (late-onset), with the latter more commonly encountered. Person-in-the-barrel syndrome is marked by bilateral brachial diplegia, intact cranial nerves, and preserved lower extremity strength. We describe the unique clinical profile of a patient with a six-week history of significant neck and bilateral upper extremity weakness who was diagnosed three months prior to the onset of these symptoms with moderately differentiated squamous cell carcinoma within the base of the tongue (Stage III T2N1M0) and metastasis to the cervical lymph nodes. She underwent concurrent chemotherapy with three cycles of cisplatin (197 mg {100 mg/m^2^} x 197 m^2^) and hyperfractionated external beam radiation therapy (total dose cGy 7000 cGy in 35 fractions {200 cGy per fraction}). She reported the rapid onset of neck and bilateral upper extremity weakness six weeks following cisplatin termination and four weeks after radiation termination. A cervical MRI suggested myositis of the cervical paraspinal muscles, and electrodiagnostic studies indicated an inflammatory myopathic process involving the cervical paraspinal and shoulder girdle muscles. The patient attained a complete resolution of her symptoms eight months after onset. This case illustrates the rare phenomenon of early-onset DHS and person-in-the-barrel syndrome caused by radiation-induced myositis. Prompt recognition of the symptoms associated with DHS and timely treatment offer the best prognosis for recovery.

## Introduction

Dropped head syndrome (DHS) refers to severe weakness of the neck extensor muscles resulting in the inability to extend the neck with subsequent chin-on-chest deformity [1-6]. This condition may result from myriad neuromuscular or extrapyramidal disorders such as amyotrophic lateral sclerosis (ALS), myasthenia gravis, inflammatory myopathy, chronic inflammatory demyelinating polyneuropathy, hypothyroidism, congenital myopathy, and Parkinson’s disease [1,2,7], however, may also be rare sequelae of radiotherapy usually for head and neck cancer, in particular, Hodgkin’s lymphoma [1,4,6,8]. DHS more commonly develops as a late complication of radiation (up to 30 years post-radiation) [1,2,8,9]. Only a few cases have been reported with early-onset DHS after radiation (within a few weeks or months) [4,10,11].

Person-in-the-barrel syndrome is characterized by bilateral brachial diplegia, intact cranial nerves, and preserved lower extremity strength [12]. We previously reported a case of person-in-the-barrel syndrome following cervical spine surgery, marked by the weakness of the deltoids, biceps, infraspinatus, and brachioradialis bilaterally postoperatively [12]. 

Herein, we present the unique case of a patient with both early-onset DHS and person-in-the-barrel syndrome following chemotherapy and radiation for squamous cell carcinoma of the tongue. The clinical and electrodiagnostic (EDX) findings are discussed. The differences in early- versus late-onset DHS, the proposed mechanism of DHS, and the diagnosis and management of DHS are also highlighted. 

## Case presentation

History, hospital course, and radiological imaging

A 64-year-old female (BMI: 34.42 kg/m^2^) reported a six-week history of significant neck and bilateral upper extremity weakness as well as dysphagia and dysarthria. She had been diagnosed three months prior to symptom onset with moderately differentiated squamous cell carcinoma within the base of the tongue, human papilloma virus (HPV) positive (Stage III T2N1M0) with metastasis to the cervical lymph nodes. She had been treated with concurrent chemotherapy with three cycles of cisplatin (197 mg {100 mg/m^2^} x 197 m^2^) and hyperfractionated external beam radiation therapy. The cisplatin was administered since the patient had a 5.0 mm nonspecific noncalcified pulmonary nodule within the right lung apex which may have been infectious, inflammatory, or metastatic by chest CT. The radiation was focused on the base of the tongue (BOT) regimen which involved a volumetric modulated arc therapy (VMAT) modulated beam and 3 arcs intensity-modulated radiation therapy (IMRT) to the base of the tongue and regional lymph nodes. The total dose administered was 7000 cGy in 35 fractions (200 cGy per fraction). 

At the conclusion of chemotherapy and radiation, the patient denied any dysarthria, dysphagia, or weakness of the neck or arms. She reported the rapid onset of neck and bilateral upper extremity weakness six weeks following cisplatin termination and four weeks after the radiation termination. Dysarthria and severe dysphagia developed within the next week. She also complained of symptoms of neuropathy in her hands and feet as well as tinnitus after being treated with cisplatin. 

Laboratory investigation one day after the initiation of the neck/arm weakness revealed a total creatine kinase (CK) level of 82 U/L (26-192 U/L) which increased to 136 U/L six days later. The patient underwent gastrostomy tube placement due to the dysphagia. She was treated with intravenous methylprednisolone (a single 125 mg injection) while hospitalized followed by an oral prednisone 10 mg taper upon hospital discharge. 

**Figure 1:**
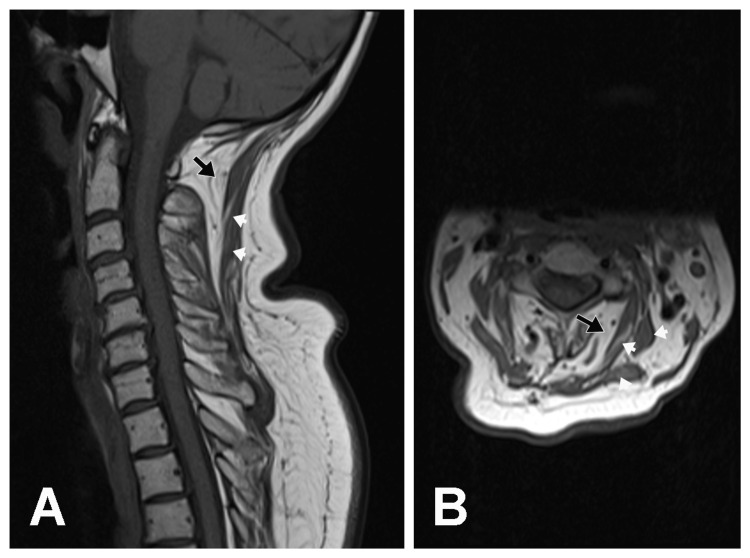
Sagittal and Axial T1-Weighted MRI Images of the Cervical Spine (A) Sagittal and (B) axial T1-weighted MRI images of the cervical spine demonstrating extensive fatty degeneration of paraspinal muscle (black arrows) and atrophic paracervical muscles containing white areas of fatty degeneration (white arrowheads).  Also note the radiation-induced fatty marrow replacement changes in the cervical spine.

**Figure 2:**
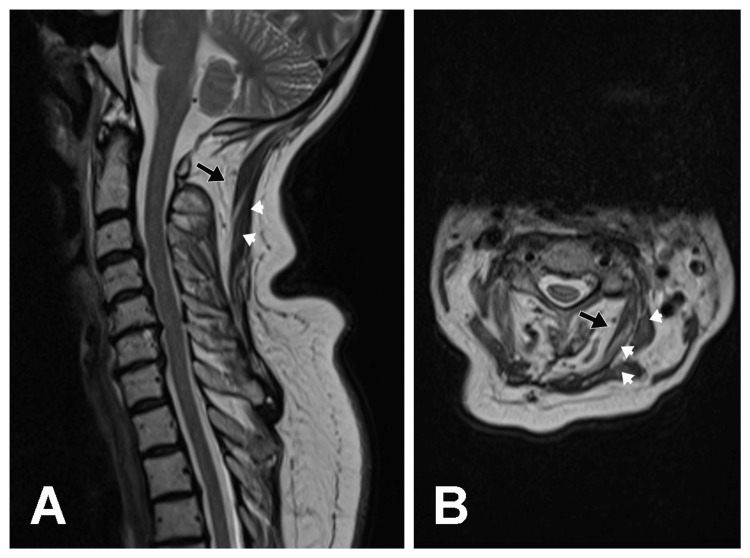
Sagittal and Axial T2-Weighted MRI Images of the Cervical Spine (A) Sagittal and (B) axial T2-weighted MRI images of the cervical spine demonstrating extensive fatty degeneration of paraspinal muscles (black arrows) and atrophic paracervical muscles containing white areas of fatty degeneration (white arrowheads).  Also note the radiation-induced fatty marrow replacement changes in the cervical spine.

T1 weighted (Figures 1A, 1B) and T2 weighted (Figures 2A, 2B) cervical MRI scans demonstrated extensive fatty degeneration of paraspinal muscles (black arrows) and atrophic paracervical muscles containing white areas of fatty degeneration extending from the skull base to the C7 level in the posterior paraspinous region, suggestive of myositis.

There was also radiation-induced fatty marrow replacement changes in the cervical spine. The brain MRI with and without Gadolinium contrast revealed no evidence of metastasis. Past medical history included pre-diabetes mellitus, hypertension, hyperlipidemia, and sleep apnea. 

Physical Examination

Both flexor and extensor muscles of the neck as well as deltoid and biceps muscles were markedly weak bilaterally. There was also weakness of the triceps muscles and intrinsic hand muscles bilaterally as well as the right facial muscles. Nystagmus to the right on right gaze and limitations of tongue movements in all directions were noted. The deep tendon reflexes of the upper extremities were hypoactive. Sensory examination was normal.

*Electromyography/Nerve Conduction Velocity* (*EMG/NCV) of the Arms*

**Figure 3:**
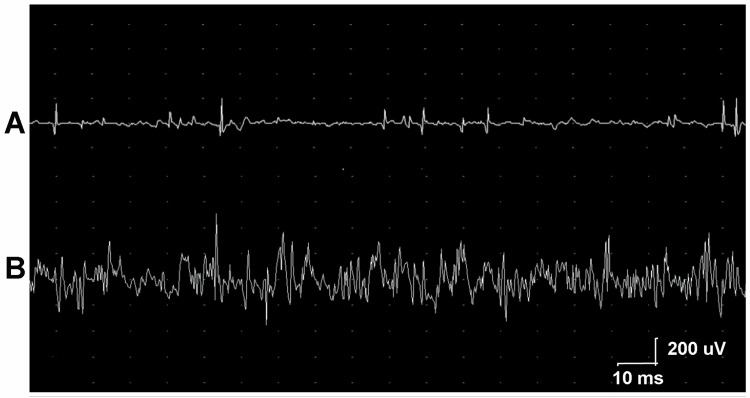
Electrodiagnostic Study of the Arms (A) Fibrillations in the right deltoid. (B) Polyphasic motor units with a short duration and small amplitude in the right deltoid.

Needle EMG revealed mostly myopathic units with profuse fibrillations, especially in the cervical and paraspinal proximal upper extremity muscles (Figures 3A, 3B).

A combination of neuropathic and myopathic changes were observed in the distal upper extremity muscles. Motor conduction was normal in the median and ulnar nerves, however, there was prolongation of sensory latency in the median nerves. The EDX studies suggested an inflammatory myopathy involving the cervical paraspinal and shoulder girdle muscles. The EMG pattern in the distal upper extremity muscles were more neuropathic and along with the sensory conduction abnormalities were attributed to chemotherapy-induced neuropathy. 

Muscle Biopsy

**Figure 4:**
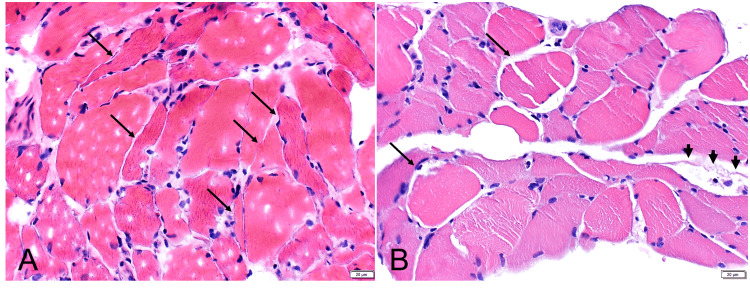
Biopsy of the Left Biceps Muscle Muscle biopsy demonstrated both denervation and myopathic features. (A) Frequent atrophic fibers with angular contours (arrows) were present, suggestive of denervation. (B) Myopathic features were also observed, including occasional swollen, hyalinized fibers with partial loss of striation (arrows) and an adjacent degenerating fiber with macrophages undergoing phagocytosis (arrowheads).

A biopsy of the left biceps muscle revealed both denervation and myopathic features, the former marked by frequent atrophic fibers of both fiber types which often exhibited angular contours and stained overly dark with esterase, and the latter characterized by scattered degenerating fibers and an increased number of cells with internal nucleia (Figures 4A, 4B).

The overall findings were more consistent with a neuropathic process seen in denervation associated with secondary pseudomyopathic changes, although the possibility of a coexisting myopathic process could not be excluded. 

Follow-Up

The patient underwent a one month course of physical therapy and rehabilitation following hospital discharge. Within four months of symptom onset, there was a modest improvement in strength and sensation as well as speech and swallowing. The patient attained a complete resolution of her symptoms eight months after onset, with an ability to raise her arms above the horizontal and hold her head erect. 

## Discussion

Only a few studies have investigated the development of early-onset DHS following radiation (Table 1) [4,10,11].

**Table 1:** Features of Early-Onset Dropped Head Syndrome DHS: dropped head syndrome; SCC: squamous cell carcinoma; IMRT: intensity-modulated radiotherapy; 3DCRT: 3D conformal radiotherapy; 5-FU: 5-fluorouracil; TPF: docetaxel, carboplatin, fluorouracil Luo et al. [10]; Smillie et al. [11]; Inaba et al. [4]

Study	Number of patients	Age (years)	Type of cancer	Radiotherapy; mean dose to neck extensor muscles/fractions	Chemotherapy	Onset of DHS after radiation (months)
Luo et al.	1	53	SCC supraglottic larynx	7580 rad at 190 cGy/fraction to primary cancer, 5580 rad at 180 cGy/fraction to regional lymphatics	Cisplatin	1
Smillie et al.	5	Pt #1: 55	SCC piriform fossa	66 Gy	None	2
		Pt #2: 62	SCC soft palate	68 Gy	Cisplatin, 5-FU, taxotere	6
		Pt #3: 52	SCC head/neck	66 Gy	Cisplatin	3
		Pt #4: 63	SCC tongue base	68 Gy	TPF, cisplatin	4
		Pt #5: 51	SCC oropharyngeal	68 Gy	TPF	5
Inaba et al.	3	Pt #1: 62	Hypopharyngeal carcinoma/esophageal carcinoma	IMRT 70 Gy for hypopharyngeal carcinoma; 3DCRT 60 Gy for esophageal carcinoma (58.5 Gy/35 fractions)	CDDP, 5-FU	5
		Pt #2: 73	Nasopharyngeal carcinoma	IMRT 70 Gy and additional 3DCRtT 10 Gy for primary disease (42.3 Gy/40 fractions)	None	6
		Pt #3: 55	Nasopharyngeal carcinoma	IMRT 70 Gy (60.9 Gy/35 fractions)	CDDP	15
Present Case	1	64	SCC tongue base	IMRT 7000 cGy (200 cGy per fraction; 35 fractions)	Cisplatin	1.5

Inaba and colleagues compared three patients with DHS after radiotherapy for head and neck cancer to nine patients without DHS [4]. The mean dose to the neck extensor muscles of the three patients with DHS was 58.5 Gy, 42.3 Gy, and 60.9 Gy, while the dose was <50 Gy in all nine patients in the control group. The onset of DHS was five, six, and 15 months in the three affected patients, respectively, following radiotherapy. These authors suggested that early-onset DHS may be due to the magnitude of the radiation dose to the neck extensor muscles, and they recommended that the radiation dose should be <46 Gy. Of the nine previously reported cases of early-onset DHS after radiation, seven were also treated with chemotherapy (Table 1) [4,10,11]. 

Radiotherapy-induced neuropathy has been described as early or late onset, with the former likely caused by an autoimmune response to the inflammation and necrosis caused by the direct effect of the radiotherapy [10,11]. Several characteristics of early-onset DHS have been reported, including a lack of atrophy of the neck extensor muscles compared to late-onset DHS [4]. Early-onset DHS is often associated with acute cervical soft tissue damage and muscle weakness due to degenerative changes [3]. The early process is usually self-limiting and may benefit from corticosteroid treatment. Alternatively, the late response may be due to vascular alteration affecting the white matter in the central nervous system, causing myelopathy and myelomalacia in the spinal cord which usually does not respond well to therapy. Late-onset DHS may involve a regenerative process, angiogenesis, and potentially irreversible changes caused by muscle fibrous degeneration [3]. 

The mechanism of radiation-induced DHS is unclear [4], however, two etiologies have been suggested including (1) myopathic due to direct radiation injury to muscles or (2) neuropathic muscle weakness secondary to lower motor neuron involvement from anterior horn cell injury [1,2]. A cervical MRI, EMG, and muscle biopsy are helpful in the diagnosis of DHS [1,2,7]. These studies may uncover a myopathic cause of DHS that is noninflammatory and isolated to the neck extensor muscle, confirming the diagnosis of isolated neck extensor myopathy. A high-signal change at the cervical extensor muscle may be observed on Short Tau Inversion Recovery (STIR) MRI in early-onset DHS, while widened cervical interspinous spaces and angiogenesis in muscles may be characteristic of late DHS [3]. A muscle biopsy may also demonstrate fiber degeneration, regeneration, and necrosis with scattered inflammatory cells [3]. 

DHS may benefit from non-surgical treatments including a cervical collar/brace, physiotherapy, massage, acupuncture, and immunoglobulin [2,7,13]. DHS developing after radiation causes extensor myopathy and anterior scar contractures with focal structural changes which often do not respond well to medications [7]. As DHS represents a severe kyphotic deformity of the cervico-thoracic spine, it may lead to vertebral compression and anterior muscle contraction. In this rare situation, surgical intervention may be warranted and involve neural decompression combined with multilevel instrumental fixation and fusion [5,7,8]. 

The present case represents the rare phenomenon of early-onset DHS resulting from radiation-induced myositis. The cervical MRI suggested myositis of the cervical paraspinal muscles, and the EDX studies indicated an inflammatory myopathy involving the cervical paraspinal and shoulder girdle muscles. The muscle biopsy was of the left biceps muscle that showed both denervation and myopathic features. Biopsy of cervical paraspinal muscles may have been more relevant for confirming inflammatory myopathy. While the two total CK levels were in the normal range in our case, this finding does not negate the diagnosis of radiation-induced myositis. Although CK levels are usually elevated in radiation myositis, they have also been reported as normal in the literature [14]. 

## Conclusions

Physicians should have a high index of suspicion of early-onset DHS and person-in-the-barrel syndrome in patients who are treated with radiation for head and neck cancer. The rapidly developing radiation-induced myositis that resulted in DHS is a unique feature of our case. Prompt recognition of the symptoms associated with DHS and timely treatment offer the best prospect for functional recovery. 
